# Gait Analysis in Neurorehabilitation: From Research to Clinical Practice

**DOI:** 10.3390/bioengineering10070785

**Published:** 2023-06-30

**Authors:** Mirjam Bonanno, Alessandro Marco De Nunzio, Angelo Quartarone, Annalisa Militi, Francesco Petralito, Rocco Salvatore Calabrò

**Affiliations:** 1 IRCCS Centro Neurolesi “Bonino-Pulejo”, Via Palermo, SS 113, C. da Casazza, 98123 Messina, Italy; angelo.quartarone@irccsme.it (A.Q.); annalisa.militi@irccsme.it (A.M.); francesco.petralito@irccsme.it (F.P.); roccos.calabro@irccsme.it (R.S.C.); 2 Department of Research and Development, LUNEX International University of Health, Exercise and Sports, Avenue du Parc des Sports, 50, 4671 Differdange, Luxembourg; alessandro.denunzio@lunex-university.net

**Keywords:** gait analysis, neurorehabilitation, neurological disorders, wearable sensors, non-wearable sensors

## Abstract

When brain damage occurs, gait and balance are often impaired. Evaluation of the gait cycle, therefore, has a pivotal role during the rehabilitation path of subjects who suffer from neurological disorders. Gait analysis can be performed through laboratory systems, non-wearable sensors (NWS), and/or wearable sensors (WS). Using these tools, physiotherapists and neurologists have more objective measures of motion function and can plan tailored and specific gait and balance training early to achieve better outcomes and improve patients’ quality of life. However, most of these innovative tools are used for research purposes (especially the laboratory systems and NWS), although they deserve more attention in the rehabilitation field, considering their potential in improving clinical practice. In this narrative review, we aimed to summarize the most used gait analysis systems in neurological patients, shedding some light on their clinical value and implications for neurorehabilitation practice.

## 1. Introduction

Gait and postural impairments are the most frequent symptoms of neurological diseases (ND), including acquired brain injury (stroke and traumatic brain injury (TBI)) as well as neurodegenerative disorders, including Parkinson disease (PD), cerebellar ataxia (CA), and multiple sclerosis (MS). These impairments often reduce the quality of life of people affected by ND, limiting the activities of daily living [1]. Altered gait patterns in stroke survivors include a decreased stance phase and prolonged swing phase on the paretic side, in addition to a reduction of walking speed and shorter stride length [2]. In TBI, the pattern of gait is quite similar to stroke, depending on the degree of injury (i.e., mild, moderate, and severe). Generally, there is a reduction in speed walking, with important difficulties in walking in tandem and in maintaining balance [3]. In neurodegenerative disorders, such as PD, gait alterations are influenced by rigidity and postural instability, both affecting the forward limb propulsion and the spatiotemporal gait parameters, including speed and step length [4]. In MS, the individuation of a single pattern of gait alteration is more complicated due to the several localizations of demyelination plaques, above all pyramidal (in 42%) and/or cerebellar (in 25%) [5]. According to Benedetti et al. [6], MS patients may show a decrease in walking speed, shorter strides, and prolonged double support intervals, accompanied by spasticity, ataxia, fatigue, and muscle weakness. All these gait abnormalities can be objectively investigated through gait analysis systems and other technologies able to capture movements during walking.

Gait analysis can be defined as the set of procedures that are needed to observe, record, analyze, and interpret human locomotion [7]. In fact, digital-based technologies are fundamental to provide kinetic, kinematic, and muscle activation information that are not detectable by clinical observation alone [8]. Observational gait assessment methods, including common rating scales, have been widely used by physiotherapists to investigate gait and balance as well as motor function in clinical practice. Clinical gait assessment investigates the person’s ability to walk and “how” they walk, considering also the fatigue level during gait [9]. For example, clinical tests for acquired brain injury patients usually include the 10-min walking test (10 MWT), Functional Ambulation Scale (FAC), or Motor Assessment Scale (MAS) [10], while in PD patients, the Unified Parkinson’s Disease Rating Scale (UPDRS) and Hoehn and Yahr scale are both used to stage the severity of the disease as well as to evaluate motor symptoms (including dyskinesia, resting tremor, muscle stiffness, and postural control) [11]. In SM patients, the Expanded Disability Status scale is commonly used to classify patients according to motor disability, whereas CA gait skills are often assessed with the scale for the Rating and Assessment of Ataxia [12,13].

**Figure 1:**
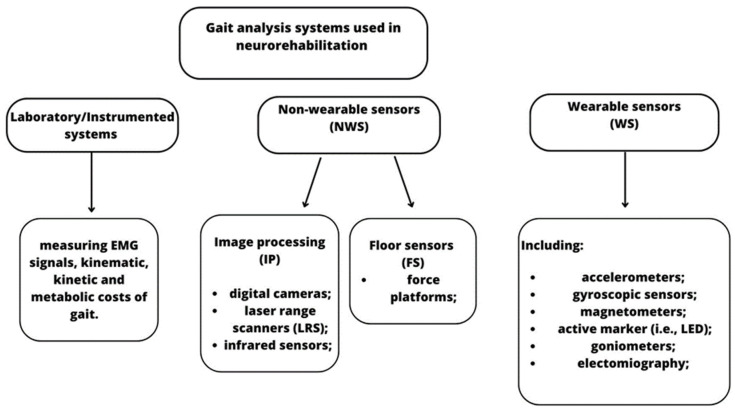
Classification of the main NWS and WS technologies used in gait analysis for neurological patients.

Despite their common use in the clinical context, these scales have been criticized since they lack specificity, responsiveness, and/or reliability, and they also require high levels of expertise from clinicians. For these reasons, observational gait assessment tools and clinical scales may be useful for unidimensional or unspecific gait evaluation, but they are not appropriate for multidimensional gait analysis that is performed by gait-related technologies. These devices are classified as non-wearable (NWS) (i.e., laboratory-based motion capture systems, plates, and platforms) and wearable sensors (WS) (i.e., magnetometers, accelerometers, and force sensors) [14] (see Figure 1).

Notably, NWS and laboratory systems are considered the gold-standard for the detection of accurate movements; however, they are expensive and are not easy to adapt to everyone, also requiring specific spaces and staff. On the other hand, WS are more suitable than NWS due to free-living gait assessment that can continuously monitor patients in their real-life setting where natural dual-tasking or social interactions occur [15].

Despite the validity and reliability of these systems to evaluate gait parameters, clinicians still question if these tools are useful in clinical practice, especially in patients affected by ND. Sometimes the high costs of instruments, infrastructure challenges, and the complexity of data discourage their use. However, a more accurate assessment could promote a tailored and personalized rehabilitation approach thanks to the collaboration between bioengineering professions and medical practitioners, including neurologists, physiotherapists, and kinesiologists.

The aim of this narrative review is to summarize the most used gait analysis systems in neurological patients, shedding a light on their clinical value and implications for neurorehabilitation practice.

## 2. Search Strategy

**Figure 2:**
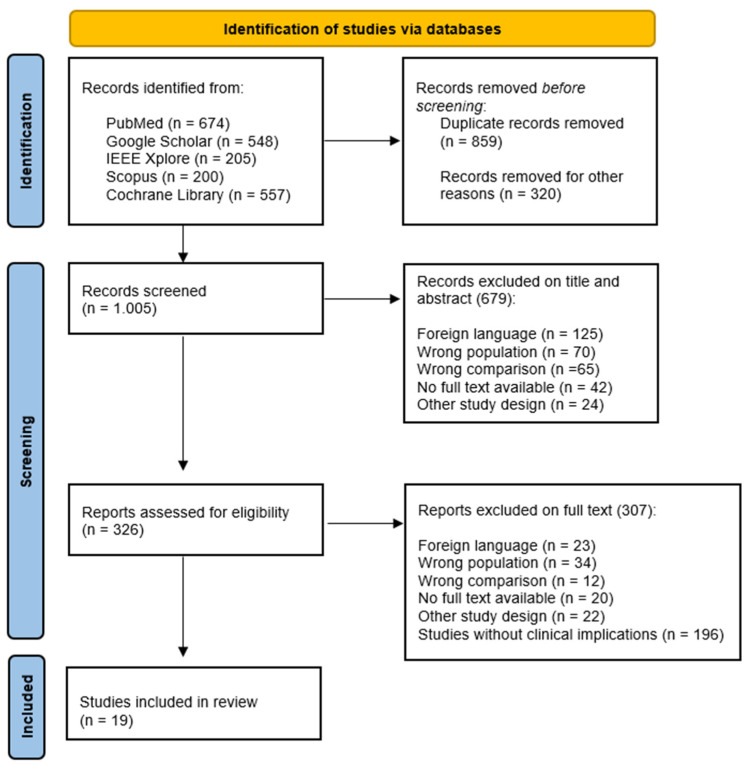
PRISMA flow chart for the study selection.

We collected evidence by searching on PubMed (Medline), Google scholar, Scopus, IEEE Xplore, and the Cochrane library from 2010 to 2023; we chose this period because this last decade has witnessed the implementation of innovative technologies used to perform gait analysis in ND. To select evidence, we used the search strategy reported in Appendix A (Table A1). Since this is a narrative review, we included the most relevant pilot studies, observational studies, randomized controlled trial, case-control studies, and systematic reviews in English language (Figure 2). Each article was evaluated through the title, abstract, text, and their scientific validity.

## 3. Neurodegenerative Disorders

Neurodegenerative disorders cause progressive neuronal loss that consequently worsens postural control and gait ability over time [16]. In this way, some specific features of gait patterns can occur for each pathology, and clinicians should consider them during the rehabilitation path because they could require different types of motor training (Table 1).

Specifically, these features (measured by sensor/instrumental technology) can be divided into three main groups: (i) spatial–temporal parameters (spatial ones include the physical distance between two steps, while temporal parameters are referred to the time used to complete a gait cycle, including cadence, duration of swing, and stance phase); (ii) kinematic parameters, which evaluate specific range of motion of ankle, knee, and hip; and (iii) kinetic parameters that measure forces involved in the gait cycle, such as the ground reaction force (GRF) [17].

### 3.1. Parkinson’s Disease

PD is one of the most common neurodegenerative disorders affecting the basal ganglia, and then, mainly the automatic gait and motor initiative. Gait alterations include (i) reduced walking speed, (ii) shorter step length, (iii) gait asymmetry, (iv) reduced arm swing, (v) loss of dissociated arm and trunk movements during gait, and (vi) postural instability [27]. Gait changes in PD patients can also occur during the early stages of the disease, affecting swing and double support time on the less-affected body side. In this way, gait parameters could be considered a biomarker of pathological progression [28]. Pistacchi et al. [19] revealed that early-stage PD patients can present slight flexion of the ankle during the swing phase, reduced dorsiflexion in the stance phase, with a mild reduction in flexion of the knee. The evaluation of these gait abnormalities is obtained through several sensors’ technology, including both WS and NWS. Specifically, the wearable inertial sensors, such as accelerometers, gyroscopes, and magnetometers, are the most used to detect kinematic and spatial temporal measures in clinical practice. According to Del Din et al. [20], accelerometers may offer more accurate information about gait variability and asymmetry than NWS (i.e., standard laboratory systems) in the PD patient population. In fact, they seem to be more sensitive in detecting gait changes due to aging and/or pathology, whereas the adjunct of the harmonic ratio (an index of harmonicity and regularity of gait) adds greater accuracy in PD gait analysis than only spatial–temporal parameters [28,29]. Other tools to perform gait analysis in such patients include motion capture systems, which represent a gold standard in detecting kinematic and kinetic parameters of human gait. However, these systems are expensive and require a lot of time and effort, and therefore, they are not suitable for home-based gait registration. For these reasons, Jakob et al. [30] developed a mobile motion capture system providing a quick and smart assessment of gait, for several hours in flexible environments. The authors demonstrated that mobile devices for gait analysis accurately registered gait speed, stride length, and stride time when compared to other capture systems. Similarly, Albert et al. [31] compared 3D full-body kinematics based on the inertial sensors/optoelectronic system and a smartphone application for the analysis of gait in PD patients. It seems that both solutions may provide detailed and clinically relevant information, although the mobile application was a more ecological and user-friendly solution, calculating stride, swing and stance phase duration, and cadence. Interestingly, Liu et al. [32] performed a quantitative analysis using an error state Kalman filter (which is a useful method to estimate the kinematic of lower limb during gait reducing joint angle drift errors) by extrapolating data from WS systems [21]. The authors found that this innovative method can recognize altered gait patterns of PD patients when compared to healthy subjects with similar demographic features, suggesting its use as an objective gait assessment tool.

### 3.2. Multiple Sclerosis

MS patients often present with gait and balance abnormalities caused by random demyelination plaques, affecting different parts of the central nervous system. The pathological gait features are related to the presence of spasticity, ataxia, muscle weakness, and sensory and proprioceptive deficits, causing a progressive disability [22]. Specifically, MS subjects can manifest a reduction in hip extension during the stance phase of gait, which may be linked to augmented tone in quadriceps and weakness of hamstrings, or also known as hip extensor muscles [23]. In the swing phase, it has been shown a reduced knee flexion, which is one of the most important gait features in these patients, and it is a valid predictor of walking function/speed. In fact, the reduced knee flexion is due to paresis of the hamstrings, increased tone in the rectus femoris, and decreased push-off power of the gastrocnemius [33]. Other important kinematic factors are related to the ankle joint. In fact, in stance phase there is a reduced dorsiflexion and limited plantar flexion during the toe-off, while in the swing phase, the plantar flexion tone tends to increase [32,33]. Altogether, these gait abnormalities can be analyzed through three-dimensional gait analysis (3DGA) using motion capture systems, divided into passive systems that need to be calibrated before the recording session (consisting in a set of multiple cameras to track 3D trajectories, i.e., Vicon, Oxford UK) or active systems that are able to identify automatically movements through infrared light-emitting diode (LED) (i.e., Optotrack, NDI, Waterloo, ON, Canada), placed on different body landmarks [9]. In this way, kinematics is calculated through marker trajectories thanks to the combination of muscle activity and external forces, such as the gravitational ones, whereas the kinetics parameters are calculated through the synchronization of kinematics and GRF. Severini et al. [24] used a laboratory-based motion capture system to collect kinematic data about gait alterations in MS individuals, showing a decreased range of motion at the hip, knee. and ankle with an augmented pelvic tilt, which is negatively correlated with a worse speed in the 6-min walking test. For these reasons, the instrumental/laboratory gait analysis, registering spatial–temporal and kinematic parameters, is a reliable tool for tracking the advancement of the disease. According to Sehle et al. [25], kinematic gait parameters can be fully evaluated through the wireless system equipped with a camera and eleven active infrared markers placed bilaterally on different body parts of SM patients (i.e., calcaneus, Achilles tendon, behind knee, ilium, and sternum). This analysis allows also to get an objective assessment to measure motor fatigue in MS patients. In fact, motor fatigue in MS tends to increase when isometric strength in the quadriceps muscle reduces its force on lower extremities during walking. These findings suggested that subjects affected by MS, as well as PD patients, manifest difficulties in coordination of reaping steps and strides and in control of the rhythmic displacements of the upper body during gait. Interestingly, Hu et al. [26] applied machine learning to gait parameters obtained by walkway sensors to distinguish MS patients as mild to moderate, according to their gait impairments severity. The authors achieved 75% accuracy in detecting gait features, with 90% precision in predicting subjects with “moderate” alterations.

### 3.3. Cerebellar Ataxia

Cerebellar ataxia (CA) consists of a group of pathologies resulting from cerebellum damage due to genetic and/or acquired causes. CA is characterized by gait disturbances that greatly affect balance and postural control. It has been shown that [34] CA patients present specific gait alterations compared to healthy controls, such as reduced walking speed and cadence, and a shorter step length, stride length, and swing phase, whereas they present an augmented base width, stride time, step time, and stance phase. Notably, the increased base width and reduced step length are considered as compensation movements related to trunk instability, while the gait abnormalities and the increased coactivation of the antagonist muscles at a single-joint level are linked to the real presence of cerebellar damage [35]. In fact, the abilities to codify multi-sensory features and to provide an “error-correction mechanism” are lost after cerebellum damage. In this context, Matsushima et al. [36] assessed gait features in CA subjects using a triaxial accelerometer placed on their back. This device is more advantageous than stabilimeter platforms or force plates since it allows registering more gait parameters, including cadence, gait speed, step length, and three-dimensional space. Additionally, it is a smart and easy solution to perform objective gait analysis in a clinical context since it does not require much time for measurements, as confirmed also by other authors [36,37,38].

## 4. Acquired Brain Injury

Post-stroke and TBI patients often show gait and balance alterations that are strictly related to the localization and extension of the brain damage [39]. In clinical practice, physiotherapists must face those abnormalities, which represent a challenging issue in neurorehabilitation. An accurate assessment of gait kinematics could be helpful to establish the degree of impairment and to plan better and more specific motor training [40]. This is why physiotherapists should not only administer observational scales, but they should use technological tools able to perform an objective analysis of gait in order to achieve better outcomes (Table 2).

### 4.1. Stroke

The gait pattern in the post-stroke population is usually characterized by temporal and spatial asymmetry between steps. In fact, temporal asymmetry in hemiparetic gait presents with a prolonged paretic swing phase on the paretic side and an augmented stance phase on the unaffected side [41]. Since post-stroke patients are a heterogenous group for the brain site and the extension of damage, they can show different paretic or nonparetic step length patterns, which are in association with compensatory adjustments of the pelvis and unaffected side [44]. Spatial parameters, such as cadence and gait speed, are also altered in the post-stroke population. Patterson et al. [45] used the GAITRite, which is a pressure-sensitive mat, to assess spatiotemporal parameters and gait symmetry in hemiparetic patients, finding that swing time, stance time, and step length asymmetries tend to get worse in the later stages poststroke. Similarly, the Strideway platform provides information about plantar pressure assessment, which is fundamental to evaluate the load distribution of the human body during walking. However, these laboratory systems or NWS technologies are rarely used in clinical context due to their high costs, non-portable systems, and need operational space. On the contrary, WS are smart, low-cost systems and can be used to assess overground gait. Indeed, Laudanski et al. [46] obtained kinematic data using inertial sensors on post-stroke patients, measuring joint angles of the pelvis, upper leg, lower leg, foot, and toe. This technology could be employed in clinical contexts to test gait patterns in post-stroke patients thanks to their quick and simple use. However, some authors suggested that a single inertial unit placed on the lower back is not often accurate in detecting spatial and temporal parameters.

Another smart and low-cost solution to perform gait analysis in post-stroke patients was provided [42] using the Microsoft Kinect (Microsoft, Redmond, WA, USA), which is an infrared camera. Notably, the second version of the device Kinect v-2 was used to collect accurately spatial–temporal and kinematics parameters of gait, showing reliability and validity in individuating different gait performance between patients and healthy controls, and between post-stroke patients with or without risk of falling. Recently, Wang et al. [43] performed gait analysis through a three-dimensional motion capture system named Odonate. This system comprises a binocular camera combined with an artificial intelligence system able to automatically capture, analyze, and process gait parameters. Compared to the passive Vicon motion capture system, Odonate showed reliability and validity in measuring spatiotemporal parameters, making it a promising tool for objective gait assessment in the clinical context.

### 4.2. Traumatic Brain Injury

Establishing gait patterns in the TBI population is extremely difficult because of the different sites and severities of brain damage (i.e., mild, moderate, and severe), and the persistence of symptoms for days/weeks (mild and acute sequelae) or for months/years (moderate/severe and chronic sequelae) [47]. In general, post-concussion patients show reduced gait speed and step length, as in post-stroke patients, PD, and SM. It has been found [48] that TBI individuals are further exposed to center of pressure (CoP) displacement alterations, with decreased velocity in both the medial/lateral and anterior/posterior planes, and also during dual task activity [49]. Belluscio et al. [50] evaluated gait alterations through three different motor walking tests (i.e., 10 min walking test, the figure-of-8 walk test, and the Fukuda stepping test) while patients were wearing inertial measurement units (IMUs). For step and stride segmentation, the IMUs were placed on the shanks and lateral malleoli, whereas to assess upper body stability, the IMUs were allocated on the occipital bone, sternum, and between the fourth/fifth lumbar vertebrae. It has been suggested that TBI subjects, during walking, need to stabilize their head, attenuating gait speed. In this light, vestibular/cervical proprioceptive training strategies should be considered during the rehabilitation path of this patient population. In a similar way, Pitt et al. [51] assessed gait and balance performances in acute TBI survivors through IMUs, demonstrating that anterolateral balance shifting is one of the most impaired motor functions up to months post-injury. According to a systematic review [47], laboratory systems and IMUs can be used to evaluate gait in TBI patients; however, the IMUs and 3D motion capture systems seem to be a smarter solution with excellent validity and reliability in detecting gait abnormalities in comparison to healthy controls.

## 5. Discussion

**Figure 3:**
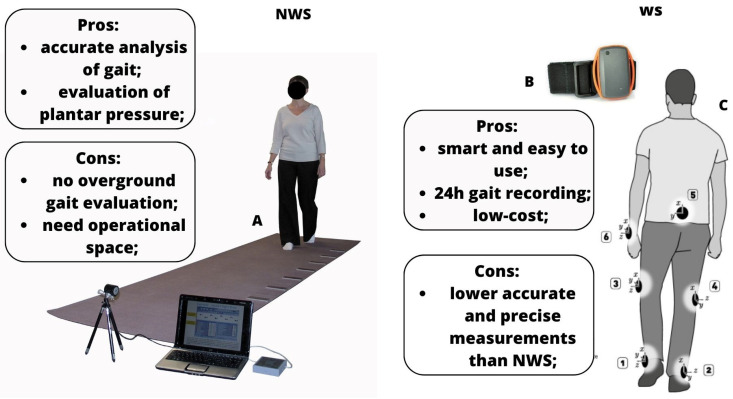
Pros and cons of NWS and WS systems ((**A**): gait analysis using NWS, like mat pressure sensors; (**B**): WS system; (**C**) (from 1–6): points in which WS can be placed to perform gait analysis).

To the best of our knowledge, this is one of the few reviews [1,52] investigating the clinical use of the main gait analysis technologies in neurological patients. From the literature, data emerges that WS, 3D motion capture systems, and IMUs are the most used gait assessment tools in the neurological population thanks to their smarter characteristics, and present less disadvantages than the NWS and/or laboratory systems (see Figure 3).

In particular, our review discusses the clinical implication and utility of using technological devices to perform gait analysis in ND, and the possible future direction of this type of evaluation in the neurorehabilitation context. The novelty of our review consists of us having considered innovative technologies used to perform gait analysis, in addition to specific clinical information about gait function, in this specific patient population. Other authors [1,2,3,4,5,6,7,8,9,10,11,12,13,14,15,16,17,18,19,20,21,22,23,24,25,26,27,28,29,30,31,32,33,34,35,36,37,38,39,40,41,42,43,44,45,46,47,48,49,50,51,52] have previously addressed only the technological field of gait analysis, without including the clinical issues related to ND and their rehabilitation approach. For these reasons, our work differs from the previous ones as we promoted a multidisciplinary approach in both evaluation and treatment for ND.

### 5.1. Clinical Considerations about Gait and Postural Dysfunctions

It is not surprising that gait and postural dysfunctions are the most prevalent motor symptoms in ND, exposing them to an increased risk of falls and subsequent hospitalization, with a great reduction in quality of life and a high risk of mortality [53]. In ND, gait stability, measured with instrument-based/laboratory systems, revealed that increased variability and asymmetry during walking are considered the most important predictive characteristics for the risk of falling [15]. Additionally, alterations in balance, as well as in the base of support, are also predictive markers of an increased risk of falling. In particular, some authors stated that 50–80% of MS patients show both balance and gait abnormalities, and over 50% reported a fall at least once each year [22]. In PD patients, walking impairments such as freezing of gait occur in 39.9%, especially in those with 10 years of the disease and with a H&Y score >2.5 [54]. Similarly, acquired brain injury patients have gait impairments in 80% of cases due to loss of postural control, cognitive alterations, and executive dysfunctions [47,55]. Traditionally, spatial–temporal parameters of gait, such as velocity, stride length, and step time, are the most quantified ones during the analysis of gait. However, alterations in spatial–temporal parameters may not be sufficient to differentiate ND. In this context, gait variability, symmetry, and coordination should be addressed too. In detail, gait variability refers to a quantifiable feature of gait that is almost impaired in ND, thus it can be used as a biomarker of pathology progression [56]. According to a systematic review and meta-analysis [56], PD and MS patients presented the lowest level of alterations in gait variability, while CA individuals showed the intermediate ones. This result in CA patients may be related to a deficit in lower limb coordination reflecting arrhythmic muscle contractions between flexors and extensors during gait. Otherwise, gait variability in post-stroke individuals was found to be augmented in stride time, which contributes to the incoordination since it directly involves the accuracy and the consistency of gait coordination [57]. In a similar way, TBI patients tend to show an increased step time variability, which is strongly correlated with dynamic instability, which further increases when the gait task becomes more challenging [58].

In the rehabilitation context, both aerobic and resistance exercise training could be useful to improve gait symmetry, weight bearing asymmetry, cadence, and step length in ND [59]. This is attributable to the increased strength in the lower paretic/affected limb that provides a greater propulsive force during gait [60]. Otherwise, balance exercises using virtual reality can further influence kinematic variables, since it has been demonstrated that postural behavior can be influenced by gait speed, direction, and sensory inputs [61]. These clinical considerations are fundamental to develop tailored rehabilitation interventions, although they require specific technological systems (such as WS and NWS) to objectively evaluate and monitor over time motor improvements in patients with ND.

### 5.2. Clinical Implications of NWS

Developments in biomechanical modelling have recently facilitated the interpretation of gait analysis reports through 3D technologies. Indeed, biomechanical models include information about skeletal anatomy, muscles, and tendons, and electromyography (EMG) activity in order to figure out the underlying mechanisms subtending gait deterioration and to potentially give explanations about the effects of specific rehabilitative interventions. In this context, Peppe et al. [62] confirmed that performing a gait analysis to quantify gain during motor training is reliable and useful in the PD clinical context. Similarly, Guner et al. [63] stated that the use of a gait analysis laboratory to evaluate the effects of yoga therapy in MS patients is impactful in revealing improvements in the spatial–temporal parameters of gait.

**Figure 4:**
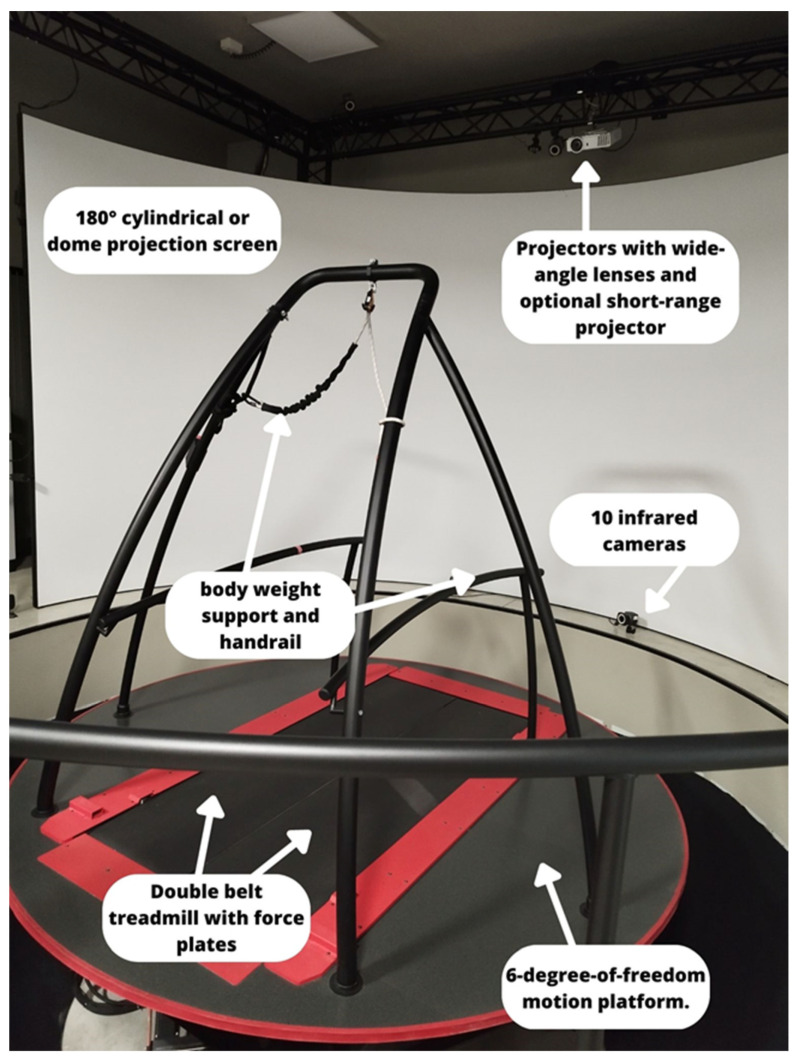
The CAREN EXTENDED system and its components, allocated at the IRCCS Neurolesi Center “Bonino-Pulejo”.

Innovative laboratory systems could include the Computer Assisted Rehabilitation Environment (CAREN) (Motekforce Link, Amsterdam, The Netherlands), which associates a split belt treadmill, a virtual reality system for rehabilitation purposes, and a Vicon motion capture system [64], allowing analysis of gait during the rehabilitation performance with an immersive virtual reality environment, also testing dual task activities (Figure 4).

Smith et al. [65] evaluated the gait cycle through CAREN, analyzing the relationship between walking speed and spatial–temporal stride parameters in healthy subjects, revealing a connection between slow walk and better foot contact on the ground. However, there is not any evidence about the use of the CAREN as a gait assessment device in the neurological population. In this context, future studies could test the reliability of the tool for gait cycle assessment, considering also walk performance during dual task virtual activities, which are crucial in PD and MS patients, and comparing them with healthy controls.

### 5.3. Clinical Implications of WS

Wearable devices are still quite new, at least for clinical neurorehabilitation practice. WS are used to track motion outside the “obsolete” laboratory for gait analysis since they can recognize motor behavior in real-time, as well as plantar pressure and step frequency [1,52]. Thus, WS allows daily gait monitoring under different conditions. Thanks to the support offered by WS, physical therapists can accurately describe gait patterns, pointing out the underlying musculoskeletal dysfunction that alters patients’ locomotion. However, in current clinical practice, WS are still not common, and physiotherapists usually rely only on clinical scales based on observational gait analysis to assess walking and balance functions in ND. According to Ferrarello et al. [39], only the GAIT scale, which specifically tests the kinematic parameters of walking, was considered as the reliable tool for clinical gait assessment in post-stroke patients. Clinical observational analysis of gait alone seems to be not enough to establish an accurate “diagnosis” of gait abnormalities, which differs among the ND etiologies. This is why the multidisciplinary synergy between rehabilitation professional figures and bioengineers could provide useful clinical information about gait assessment in ND. In fact, the objective nature of gait assessment through WS and its practical advantages (Figure 2) in the clinical rehabilitation setting should motivate its adoption. Nevertheless, it should be considered that WS have some limitations that may be related to (i) the amount of time requested to analyze and review all the collected data, (ii) privacy and security issues, and (iii) technological equipment that requires high skilled professional figures [1,52].

Other kinds of wearable devices used in neurorehabilitation motor training include robotics (i.e., end-effectors and/or exoskeletons), which could be used to automatically perform a sort of gait analysis to register the improvements before and after the training in spatial–temporal, kinematic, and kinetic parameters of gait. Imoto et al. [66] developed a new and valid gait training robot named Welwalk WW-2000 (WW-2000, Toyota Motor Corporation, Aichi, Japan), which is equipped with sensors and a marker-less motion capture system for hemiparetic individuals. The robot can register a patient’s gait parameters and provide tailored gait training, adapting to the patient’s gait characteristics.

### 5.4. Future Directions of Gait Analysis

Moreover, other innovative tools are paving the way for a further objective and simpler approach to gait analysis through machine learning (ML) [67]. ML is a branch of artificial intelligence (AI) that enables computers and devices to learn from data. In particular, ML methods can be applied to gait studies, focusing on the prediction of early therapeutic intervention due to fall-related risks, or on determining motor recovery tasks [68]. Specifically, ML methods applied to gait analysis proceed through dataset inputs divided into a training set, testing set, and validation set. When a ML technique is selected, the model is trained to use a specific training set and it is validated to determine the level of fitting. Lastly, the model is further tested through an unseen test dataset. When the accuracy is acceptable and the process ends, the model returns to the training phase until it reaches the level of accuracy needed [69]. In gait application, three ML techniques are commonly used: supervised, unsupervised, and reinforcement learning. Notably, supervised learning algorithms (i.e., random forest, neural network, support vector machine, etc.) try to convert feature vectors (inputs) into labels (outputs). This approach can be applied to gait analysis in order to detect muscle activity and gait asymmetry in ND. In this context, Fricke et al. [70] examined the ability of three ML algorithms to classify neurological patients, including PD, CA, and MS. They found that a type of neural network was the best to recognize neurological patients, based on EMG data, allowing a classification accuracy of 80 to 90%, even in small samples. Similarly, Liuzzi et al. [71] provided an estimation using ML to detect important information on dynamic balance and gait adaptability in ND to aid clinicians to identify clinical features that need to be improved in the rehabilitation setting.

Unsupervised learning is used to cluster ND to determine gait activities and does not use labels, but the algorithm itself makes relationships between inputs and outputs. Among ML paradigms, reinforcement learning aims to perceive and interpret the environment, taking actions and learning through its trials and errors. Its current use in neurorehabilitation includes robotics, as they can provide the ability to learn tasks [72,73,74].

Indeed, some authors investigated the role of deep learning (DL) in the context of neurorehabilitation. DL techniques can be used to detect gait features in ND, as reported by Romijnders et al. [75]. These authors validated a DL-based approach in a cohort of ND that was useful to extract data about stride-specific gait parameters from IMUs. Additionally, Kaur et al. [76] developed a DL approach to classify PD and MS patients based on gait dynamics, extracting 3D body frameworks from the recorded gait videos. This approach could be useful in the field of telemedicine as a potential in-home gait monitoring tool since alterations in gait function can be a biomarker of pathology progression.

**Figure 5:**
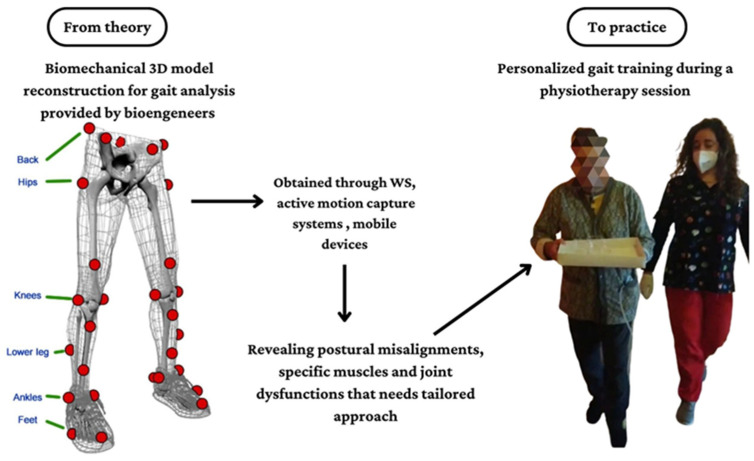
Theoretical paradigm about the use of innovative technologies to perform gait analysis in the neurorehabilitation context.

In a future perspective, innovative technologies, such as WS, ML, as well as some robotic systems, could motivate the implementation of gait analysis in the clinical context as they are getting simpler, quicker, and easier to interpret than the old methods, including laboratory systems. However, to date, there are few studies with a practical clinical implication about performing gait analysis in the neurological population, especially in CA, MS, and TBI patients, and this may be related to practical issues, such as the absence of specific devices that are not so commonly diffused in hospital settings, and economic issues. Moreover, the lack of direct collaboration between clinicians and bioengineering professional figures should be also considered. Technological development could overcome these concerns promoting the use of smart tools (i.e., smartphones), that could be frequently used in the clinical context, as confirmed by some authors [18,19]. Lastly, physiotherapists and kinesiologists could collaborate more with bioengineers to share bidirectionally practical information, which is needed to plan specific motor training based on functional findings (see Figure 5).

## 6. Conclusions

To sum up, the assessment of gait in ND requires an objective examination provided by specific instruments, such as NWS and WS technologies, that should be chosen according to patient features and etiology. In particular, WS systems such as accelerometers offer more accurate information about kinematic and spatial–temporal parameters than NWS. Therefore, WS could be more useful in the PD population as they manifest asymmetric gait with decreased step length and gait velocity. Additionally, WS, especially the accelerometer, seem to be suitable tools to detect alterations in cadence, gait speed, and step length in those affected by CA. Otherwise, NWS devices, such as the instrumented laboratory equipped with a pressure mat, are useful to measure kinetic GRFs that reveals plantar pressure distribution of the foot since it can be distinctive in SM and post-stroke patients. In TBI patients, the registration of further kinetic factors such as CoP is important for detecting alterations in balance and postural control thanks to the use of forces plates or pressure sensors. Lastly, the future of gait analysis could be simpler, quicker, and easier than previous experiences thanks to the use of innovative technologies (i.e., WS, ML, and robotic devices). Data collected from these devices could improve the quality of gait assessment and the strength of research results in the neurorehabilitation field. Thus, collaborations between researchers, bioengineers, and medical practitioners become necessary to better identify the gap between clinical necessities in the physiotherapy field and theoretical patterns of motion.

## Figures and Tables

**Table 1:** The technologies used to perform gait analysis in PD, MS, and CA, and their clinical implications, revealed by the selected studies.

Reference n°	Gait Analysis System		Technology Description	Neurological Disorder	Clinical Implication
	Non- Wearable Sensors	Wearable Sensors			
[15]	X	X	Three-dimensional gait analysis in laboratory, including optometric system, a dynamometric platform, and ad hoc software.	PD with 1.5–2 H&Y stage	Reduced gait speed and step length, showing bilateral extra rotation of knee, ankle, and foot.
[16]		X	Triaxial accelerometer-based device placed on the fifth lumbar vertebrae and a double-sided tape.	PD with 1–3 H&Y stage	NA
[17]		X	Instrumented force-sensitive insole placed in patients’ shoes, with eight pressure-sensitive sensors.	PD with 2–3 H&Y stage	Stride-to-stride variability due to bradykinesia, loss of muscle synergies in the lower limb, and lack of rhythmicity.
[18]	X	X	Motion-capture based gait analysis compared to mobile sensor (inertial sensors) gait analysis, which were integrated in the mid-sole of the athletic shoes.	PD with 1–4 H&Y stage	Reduced gait speed, stride time, and length; increased duration stance phase time accompanied by a synchronic decreasing duration of swing phase time.
[19]	X	X	Gait assessment through an optoelectronic (48 retroflected markers), inertial, and a smartphone-based capture system.	PD with <3 H&Y stage	NA
[20]		X	Wearable device compared to Opti Track system, using an error state Kalman filter algorithm.	PD	NA
[21]	X		Stereophotogrammetric system (Vicon Motion Systems Ltd., Oxford, UK) and reflective markers to estimate joints’ angles.	MS with a score of ≤5–6	MS patients showed reduced gait speed, which correlated with a decrease in cadence, step length, and swing time, and an increase in stance time. Additionally, authors found an increased pelvic tilt, which negatively correlates with the 6MWT.
[22]	X	X	Wireless AS200 system, comprising three line-scanning camera system and 11 active infrared markers attached on body’s patient, with a 2-mm accuracy.	MS with a mean score of 3.6 in EDSS	MS patients manifested changes in variability of movement gait patterns due to fatigue, altered motor coordination linked to additional activity of the antagonists, or insufficient strength produced by the agonists.
[23]	X		Walkway sensor and machine learning (XGB) process to distinguish MS patients’ degree of severity based on their gait features.	MS with a mean score of 2.11 in EDSS	Step time and step width were considered as the most important variables to distinguish level of severity of MS subjects.
[9]	X	X	SMART-E stereophotogrammetric system (BTS, Milan, Italy) with eight infrared cameras (for acquiring kinematic data). Sensorized pathway with 2 piezoelectric force platforms (for acquiring kinetic data), 22 retro-reflective spherical markers for lower-body segments, and 15 markers for the upper body, placed on specific anatomic sites.	Spino-CA autosomal dominant (type 1 and 2) and Friedreich’s ataxia as recessive ataxia	Loss of lower limbs control during gait and of ability to stabilize a walking strategy over time. CA patients definitively lack a stable gait control behavior since the cerebellum functions of motor behavior and developing new motor patterns are altered.
[24]		X	Triaxial accelerometer.	Spino-CA with a mean score of 3.9 for stance and gait in SARA	Gait velocity, cadence, step length, step regularity, and step repeatability are strongly correlated with disease duration.
[25]		X	Seven inertial sensors while performing two independent trials of gait and balance assessments.	CA	NA
[26]		X	Three Opal inertial sensors were attached on both feet and the posterior trunk at the level of L5 with elastic Velcro bands.	Spino-CA with a mean score of 3.6 for stance and gait in SARA	Minimal changes in gait spatial–temporal parameters can be considered as accurate markers for CA progression.

Legend: PD (Parkinson’s disease), H&Y (Hoehn and Yahr scale), EDSS (Expanded Disability Status Scale), 6MWT (6-Minute-Walking Test), SARA (Scale for the Assessment and Rating of Ataxia).

**Table 2:** The technologies used to perform gait analysis in post-stroke and TBI patients and their clinical implications, revealed by the selected studies.

Reference n°	Gait Analysis System		Technology Description	Neurological Disorder	Clinical Implication
	NWS	WS			
[38]	X		A 10 m walkway with a pressure sensitive mat. Spatial–temporal parameters were registered using GaitRite mat, which contains a total of 13,824 sensors.	Post-stroke patients (both ischemic and hemorrhagic)	Most useful gait parameters are step length, swing time, and stance time. In addition, authors stated that asymmetry time values are not reliable parameters to assess gait in post-stroke patients.
[39]		X	Inertial Measurment Unit (IMU) system (Xsens Technology B.V., Enschede, The Netherlands, Hengelo) composed of seven inertial sensors.	Post-stroke patients	NA
[40]		X	Kinect v2, which included an 8-core Intel^®^ in addition to an ad hoc application designed to register the 3D position and orientation of the 25 human joints provided by the Kinect v2.	Post-stroke patients (both ischemic and hemorrhagic)	Results indicated that patients with a higher fall risk manifested lower gait velocity and cadence, a shorter stride and step length, and higher double support time. Additionally, the risk of falling was related to increased trunk and pelvic obliquity and tilt, and to decreased hip flexion–extension and ankle height variation.
[41]	X		Odonate 3D motion capture system in a mobile terminal and a workstation. This innovative a binocular depth camera combined with an artificial intelligence system to capture, analyze, and calculate gait parameters automatically.	Post-stroke patients	Alterations were found in spatial–temporal and kinematic parameters; thus, this new system can perform an objective gait assessment in five minutes, also in a home-based setting.
[42]		X	Five synchronized IMUs.	Severe TBI patients	Severe TBI patients present serious difficulties in maintaining balance during gait, especially movements of the head, which are the most impaired, probably related to vestibular dysfunctions due to traumatic events. Additionally, authors suggested to assess gait through dynamic balance skills during curved trajectories as in Figure-of-8 Walk Test.
[43]		X	Three IMUs were attached with elastic straps over both lateral ankles to detect gait phases and over the fifth lumbar vertebrae.	TBI	TBI patients manifest great imbalances in dynamic balance, especially in antero-medial weight shifting, when compared with healthy control subjects.

## Data Availability

Not applicable.

## Appendix A

**Table A1:** Search algorithm used to collect studies.

**Neurological Disorders** **AND**	**Gait Analysis Systems**
“Parkinson’s disease” OR “multiple sclerosis” OR “cerebellar ataxia” OR “ataxia” OR “neurodegenerative disorders” OR “acquired brain injury” OR “stroke” OR “traumatic brain injury”	“wearable sensors” OR “gait platforms” OR “non-wearable sensors” OR “instrumented gait analysis” OR “objective gait evaluation” OR “inertial measurement units” OR “motion capture systems” OR “mobile application” OR “artificial intelligence”
